# Echocardiography in Pulmonary Arterial Hypertension: Is It Time to Reconsider Its Prognostic Utility?

**DOI:** 10.3390/jcm10132826

**Published:** 2021-06-26

**Authors:** Ioannis T. Farmakis, Eftychia Demerouti, Panagiotis Karyofyllis, George Karatasakis, Maria Stratinaki, Dimitrios Tsiapras, George Athanassopoulos, Vassilios Voudris, George Giannakoulas

**Affiliations:** 1 Cardiology Department, AHEPA University Hospital, Aristotle University of Thessaloniki, PC 54621 Thessaloniki, Greece; itfarmakis@gmail.com (I.T.F.); g.giannakoulas@gmail.com (G.G.); 2 Non-Invasive Cardiology Department, Onassis Cardiac Surgery Center, PC 17674 Athens, Greece; georgekar2001@yahoo.com (G.K.); dtsiapras@hotmail.com (D.T.); gapostol@otenet.gr (G.A.); 3 Invasive-Cardiology Department, Onassis Cardiac Surgery Center, PC 17674 Athens, Greece; pakar768@yahoo.gr (P.K.); vvoudris@otenet.gr (V.V.); 4 Department of Cardiology, General Hospital Venizeleio, PC 71409 Irákleion, Greece; maria.stratinaki@gmail.com

**Keywords:** echocardiography, pulmonary arterial hypertension, right ventricle

## Abstract

Pulmonary arterial hypertension (PAH) is characterized by an insult in the pulmonary vasculature, with subsequent right ventricular (RV) adaptation to the increased afterload that ultimately leads to RV failure. The awareness of the importance of RV function in PAH has increased considerably because right heart failure is the predominant cause of death in PAH patients. Given its wide availability and reduced cost, echocardiography is of paramount importance in the evaluation of the right heart in PAH. Several echocardiographic parameters have been shown to have prognostic implications in PAH; however, the role of echocardiography in the risk assessment of the PAH patient is limited under the current guidelines. This review discusses the echocardiographic evaluation of the RV in PAH and during therapy, and its prognostic implications, as well as the potential significant role of repeated echocardiographic assessment in the follow-up of patients with PAH.

## 1. Introduction

Pulmonary arterial hypertension (PAH) is characterized by progressive proliferation and remodeling in the pulmonary vascular bed. The insult of the pulmonary vasculature leads to an increase in pulmonary vascular resistance (PVR), with subsequent adaptation of the right ventricle to the increased afterload [1]. Ultimately, the exhaustion of compensatory mechanisms results in right ventricular (RV) dysfunction and right heart failure (RHF)—the leading cause of death for PAH patients.

The awareness of the importance of RV function in PAH has increased considerably, as it determines the functional status, exercise capacity, and outcome of patients. Non-invasive imaging techniques—such as echocardiography and cardiac magnetic resonance (CMR)—are of paramount importance in the evaluation of the right heart. Given its wide availability and low cost, echocardiography is appealing to both PAH patients and their attending physicians. According to the latest ESC/ERS Guidelines, the utility of echocardiography has been sized down to two echocardiographic parameters—the right atrium end-systolic area, and the presence of pericardial effusion—which have been incorporated in the routine risk assessment of the PAH patient [2]. However, many echocardiographic indices have been reported to have prognostic implications, and are increasingly incorporated into clinical practice; however, it is not yet clearly elucidated which exact echocardiographic parameters are imperative in the assessment of RV maladaptation and failure and, therefore, specific recommendations do not exist. This raises the interesting question of which aspects of RV function evaluated by echocardiography should be routinely used as parameters in PAH patients’ risk assessment at baseline assessment and during follow-up.

This review will discuss the pathophysiological response of the RV in PAH, as well as the prognostic implications of its dysfunction, the echocardiographic evaluation of the RV in PAH at baseline and after targeted therapy—and its prognostic implications—and, lastly, the potential significant role of repeated echocardiographic assessment in comparison with right heart catheterization (RHC) in the follow-up of patients with PAH.

## 2. Right Ventricular Function in PAH

### 2.1. Pathophysiology of Right Ventricular Adaptation and Right Heart Failure

PAH represents a progressive disorder affecting the whole cardiopulmonary unit, consisting of the right ventricle and the pulmonary vasculature [3]. These two main functional subsystems have unique intrinsic characteristics. On the one hand, load-independent intrinsic characteristics—such as ventricular contractility and diastolic stiffness—delineate the ventricular component, while, on the other hand, vascular resistance and compliance are load-dependent and describe the pulmonary vascular system [4]. The interaction between the two results in system parameters, which describe global function (ventricular and load), and these are represented mainly by (a) the ejection fraction (EF) and cardiac output (CO), and (b) the pulmonary pressure, respectively [1,4]. The term “ventriculoarterial coupling” describes the adaptation of the RV to the increased pulmonary vascular load, and is used as a measure of the efficiency of energy transfer from the right ventricle to the arterial vessels, while it has additional prognostic significance [5]. The gold standard measure for describing ventriculoarterial coupling is the E_es_/E_a_ ratio, where E_es_ indicates the end-systolic elastance—an intrinsic measure of RV contractility—while E_a_ indicates the arterial elastance, a measure of total load in the pulmonary vasculature. Both indices are estimated invasively from the pressure–volume loop [6].

The RV adaptation is a spectrum ranging from well-adapted to maladapted RV function, and progresses in relation to the increased vascular load [7]. At initial stages, the ventriculoarterial coupling is minimally altered through an adaptive remodeling, which encompasses cardiac muscle hypertrophy, resulting in a normal or mildly decreased RV function, and a preserved or mildly depressed exercise capacity and ventilatory efficiency. However, the increasingly growing pressure load leads to a halt of the hypertrophic process and reduced right cardiac function. In order to maintain the cardiac output, the heart rate increases and ventricular dilation occurs, which leads to greater oxygen consumption with less efficiency, increased RV wall stress and stiffness, and deleterious impact on the left ventricular function. Indirect left ventricular (LV) dysfunction is present in the late stages of disease, through ventricular interdependency [8]. The impaired filling of the LV owing to the leftward septal bowing and the reduced stroke volume (SV) of the right ventricle causees atrophic remodeling of the LV [9,10]. The atrophic LV has decreased contractile power, owing to the reduced cross-sectional area of the LV cardiomyocytes [11]. Experimental data have shown reverse of the LV atrophy with alleviation of the RV pressure overload, and clinical studies indicate partially restored LV function with improved hemodynamics, owing possibly to the resynchronization of the RV and LV [12,13,14].

Consequently, the maladaptive remodeling of the RV results in “uncoupling”, which is characterized by moderate or severe RV dilation, with subsequent systolic dysfunction [15]. As the disease progresses, the compensatory mechanisms are progressively exhausted, leading to uncoupling and, ultimately, to RHF, which represents a clinical syndrome that includes decreased RV function, leading to insufficient cardiac output and elevated filling pressures.

### 2.2. Prognostic Significance of Right Ventricular Function in PAH

RHF is the predominant cause of death in PAH patients [4]. This has been a consistent finding among large cohorts, but also smaller studies, where the survival of PAH patients is strongly related to the avoidance of RHF. Several registries underline the utility of right heart and pulmonary hemodynamics for survival prediction [16,17,18,19]. A common characteristic of the risk prediction models of survival in PAH is that they do not incorporate the crucial transition from RV adaptation to RV maladaptation and, subsequently, to RV failure. However, this transition is progressive, and the definition of RV maladaptation is elusive. There is ample evidence that the RV end-diastolic volume (RV EDV), the end-systolic volume (ESV), the SV, and the right ventricular ejection fraction (RVEF)—as defined by SV/EDV—contain the foremost prognostic information. In case of an adapted RV, increased afterload leads to hypertrophy and increased contractility, with preserved or mildly increased RV dimensions and preserved SV, whereas in maladaptation the RV volumes increase and the SV decreases.

Several studies have showcased the usefulness of the CMR-measured RVEF, with values < 35% being consistently prognostic of decreased survival [5,15,20]. Moreover, Vanderpool et al. showed the prognostic relevance of ventriculoarterial decoupling in 50 patients with PAH [5]. A subsequent study by Brewis et al. was able to confirm that the SV/ESV ratio predicted survival [15]. Interestingly, the SV/ESV and the RVEF share a non-linear relationship, and SV/ESV may be a better predictor of outcomes than RVEF [21]. In addition, other CMR studies have demonstrated the predictive value of RVEF, EDV index, ESV index, and RV mass index [22].

Ventricular interdependency and the subsequent LV dysfunction also have prognostic implications [23]. Indeed, LV systolic strain is reduced in subjects with PAH, and is independently associated with mortality, despite the preservation of LVEF [24,25,26]. Furthermore, the LV peak filling rate is altered in proportion to the decreased RVEF, denoting diastolic dysfunction of the LV in progressed PH [27]. Metrics of diastolic dysfunction—such as the peak early diastolic velocity and abnormal E wave velocities, as assessed by CMR tissue phase mapping—have been associated with decreased survival [28,29].

## 3. Echocardiographic Evaluation of Right Ventricular Function and Its Prognostic Relevance in PAH

The American Society of Echocardiography Guidelines offer a comprehensive review of right heart evaluation [30,31]. Estimation of RV contractility by echocardiography is a challenging task due to its unique anatomy; however, many indices have been described as parameters of RV global function. In everyday clinical practice, the most commonly used indices in PAH patients are: RV dimensions, tricuspid annular plane systolic excursion (TAPSE), RV fractional area change (FAC), eccentricity index, RV myocardial performance index (MPI), tissue Doppler imaging (TDI)-derived tricuspid lateral annular systolic velocity (RV S’), right atrial end-systolic size, and pericardial effusion [32,33,34,35,36].

TAPSE is mostly regarded as a surrogate for RV systolic function. It was found to predict survival in 63 patients with PAH [37], but failed to predict mortality in a subsequent study of 777 patients with precapillary PH—and especially those in New York Heart Association functional class III–IV with RV dilation [38]. Although TAPSE is easy to measure and reproducible, it predominantly mirrors longitudinal RV function, is volume- and load-dependent, and is not accurate in patients with regional RV wall abnormalities and significant secondary tricuspid regurgitation [39]. RV FAC reflects both longitudinal and radial components of RV contraction, correlates well with RVEF via MRI, and has been shown to predict survival in patients with PAH [38,40]; however, it neglects the contribution of the RV outflow tract to overall systolic function, and has only fair interobserver reproducibility [31]. The eccentricity index is acquired from the short-axis view of the LV. In severe PH and increased RV pressures, the interventricular septum shifts towards the left side, giving the LV its characteristic D-shape, and has been shown to add to the prognostic stratification of patients with PAH [34]. RV MPI, also known as the Tei index, incorporates systolic and diastolic time intervals to reflect global RV function [41], with values of ≥ 0.64 [38] and > 0.88 [42] reported to predict survival; however, it requires the acquisition of images in two different cardiac cycles, making it difficult to obtain, and also has not been shown to have a strong correlation with CMR-derived RVEF—as opposed to TAPSE [43]. TDI RV S’ is a reliable and reproducible technique, with the advantage of being validated by a large, population-based study [44]. Values of S’ < 10 cm/s raise the suspicion for abnormal RV function. Moreover, the isovolumetric peak velocity at the tricuspid annulus—which can be assessed by DTI—is an independent predictor of overall survival in patients with severe PH [45]. RA size and pericardial effusion use in PAH are discussed later in this review.

The TAPSE/pulmonary arterial systolic pressure (PASP) ratio seems to represent a significant marker of ventriculoarterial coupling. In patients with left heart failure, the TAPSE/PASP ratio, when combined with exercise ventilation, was a strong predictor of major cardiac events [46]. Tello et al. showed that the TAPSE/PASP ratio also has prognostic relevance in patients with PAH, and is independently associated with overall mortality, even after adjusting for other echocardiographic or hemodynamic prognostic indicators [47]. The TAPSE/PASP ratio was also validated as a surrogate of invasively measured ventriculoarterial coupling in severe PH [48]. In recent years, several other echocardiographic indices of RV function have been proposed to reflect ventriculoarterial coupling in PH patients, including the ratio of RVFAC to mean PAP (measured by right heart catheterization) [49], the ratio of RV area change to RV end-systolic area [50], the ratio of S’/RV to end-systolic area index [51], and the ratio of TAPSE to pulmonary artery acceleration time [52]. However, none of these proposed echocardiographic surrogates, with the exception of the TAPSE/PASP ratio, have been directly compared with pressure–volume loop measures of ventriculoarterial coupling. Therefore, the TAPSE/PASP ratio is a straightforward, promising echocardiographic parameter derived from routinely measured indices, fully applicable on the daily basis routine.

In addition to the traditional and well-established direct and indirect measures of RV function, a number of novel unconventional echocardiographic techniques have been introduced in the recent years. Two-dimensional (2D) speckle tracking echocardiography (STE)-derived RV strain is a measure of myocardial deformation, and is useful in the evaluation of the function of the right heart, as it is independent of endocardial border tracings and geometric assumptions [53]. The systolic function of the RV relies heavily on its longitudinal contraction, acting more or less like a piston pump; thus, the longitudinal strain—rather than the circumferential or radial strain—is the preferable parameter to measure [54]. RV longitudinal systolic strain (RV LSS) is a very useful marker of subclinical deterioration, even before conventional measures—such as the TAPSE—deteriorate. Notably, pressure overload in PAH results in the hypertrophy of the RV and, therefore, strain acquisition in the normally thin-walled RV is easier in the PAH patient than in the normal RV. In chronic PH, 2D-STE and 3D-STE parameters perform better than other conventional echo indices, such as the TAPSE and FAC, in recognizing global and regional RV dysfunction, which is associated with hemodynamic signs of RV failure [55]. Studies indicate that the RV LSS correlates well with markers of functional assessment, and is a powerful predictor of survival in PAH [56,57]. Moreover, a recent meta-analysis confirmed that the global RV LSS measured using 3D and 2D echocardiography has potential as a predictor of survival in patients with PH [58]. Recently, RV-strain-derived post-systolic patterns that reflect the RV diastolic function have been identified to be clinically meaningful and increase the prognostic power for clinical worsening [59].

Regional heterogeneity of RV function can be echocardiographically evaluated by RV dyssynchrony, which is measured using 2D-STE, and is defined as the R–R-corrected standard deviation of the times to peak systolic strain for the four mid-basal RV segments [60]. RV dyssynchrony has been found to be impaired even in mild/borderline PH, and may reflect early ventriculoarterial uncoupling [61]. The addition of RV dyssynchrony to multivariate models improves prediction of clinical worsening and decline in exercise capacity, even adjusting for conventional clinical, echo, and hemodynamic parameters [62,63].

Moreover, real-time 3D echocardiography is a promising tool to quantitate right heart chamber volumes and estimate RV function, because it captures the complex RV morphology, and compares fairly well with CMR, while normal reference values are available [64,65,66]. Studies implementing 3D echocardiography have shown adverse remodeling of the RV and the tricuspid valve in PH, which is linked to an adverse clinical outcome [67,68,69]. Furthermore, a 3D echocardiographic estimated RVEF has been shown to predict outcomes in PH [70]. 3D echocardiography techniques could be deployed in the future to produce less invasive pressure–volume analyses in order to accurately evaluate RV function [71]. However, to date, these novel methods have not been widely implemented in routine practice, as there are drawbacks—mainly related to the quality of the image acquisition; they are heavily dependent on the anatomy of the RV—the greater the diameter of the RV, the less reliable is 3D echocardiography’s capacity to calculate volumes. Thus, these indices are not always easy to obtain in all PAH patients, and are mainly used in selected patients and in expert centers for research purposes.

## 4. Echocardiography in PAH

### 4.1. The Use of Echocardiography for Risk Stratification in PAH

According to proceedings from the 6th World Symposium on PH in 2019, PAH patients should be stratified as low, intermediate, or high risk for annual mortality, at baseline assessment and during their routine follow-up every 3–6 months [72]. Several risk assessment scores have been employed in recent years [73]; among these, the ESC/ERS stratification score has been a posteriori validated by three retrospective studies, and includes clinical, imaging, laboratory, cardiopulmonary exercise testing, and hemodynamic parameters [74,75,76]. The United States Registry to Evaluate Early and Long-Term PAH Disease Management (REVEAL) score is prospectively validated, and includes multimodal parameters [18,77]. Both scores’ common goal is to direct PAH therapy and drive patients into the low-risk zone of the disease.

Of echocardiographic parameters, only the presence of pericardial effusion and the RA size are quoted for risk stratification in the aforementioned scores and current guidelines. Truly, pericardial effusion is one of the most reported prognostic parameters associated with mortality in PAH. Pericardial effusion in PAH is driven by increased right atrial pressure, which impairs the venous and lymphatic drainage of the myocardium and is, therefore, a reflection of RV diastolic dysfunction [78,79]. Pericardial effusion was reported in 54% of patients with severe IPAH, with larger effusions being associated with hemodynamic and echocardiographic evidence of right heart failure, impaired exercise tolerance, and poor prognosis at the one-year follow-up [80]. However, pericardial effusion presents late in the course of the disease—a finding which may necessitate immediate treatment with intravenous epoprostenol, as these patients present high annual mortality rates. Thus, pericardial effusion is not a frequent finding during a close patient’s follow-up, and certainly is not useful for a more refined monitoring of right ventricular function. Moreover, in certain entities—such as connective tissue diseases—pericardial effusion may reflect the serous involvement of the pericardium rather than the severity of the pulmonary vascular disease.

RA size, as an indirect measure of RV function, has also proven to be relevant to the prognosis, and in a recent meta-analysis the risk of all-cause mortality increased by 50% for every 5-unit increase in RA area [81]. In addition, metrics of RA function—such as the RA function index (RAFi) and the RA peak longitudinal strain—have been shown to strongly predict clinical failure in precapillary PH [82,83].

Although we have mentioned several other echocardiographic parameters that assess the global function of the right ventricle, and that may have prognostic relevance, it is interesting that none of them is mentioned in the current guidelines. The problem lies in the fact that the majority of these echo indices has been prognostically evaluated only by small, single-center studies, and there has not been any systemic evaluation of the RV function along with the other parameters of clinical scores. A large prospective cohort study that will simultaneously assess a wide range of echocardiographic, but also clinical, laboratory, and hemodynamic indices, is needed in order to better incorporate echocardiography in PAH risk stratification [84].

### 4.2. Effects of PAH-Targeted Drug Therapy on the Right Ventricular Function

Management of PAH has substantially improved in the last decade, and this has clear effects on long-term efficacy measures [85,86]. Phosphodiesterase type V inhibitors (sildenafil and tadalafil) and endothelin receptor antagonists (ambrisentan, bosentan, and macitentan) form the forefront of therapy in PAH, with increasing rates of upfront double combination therapy, while parenteral prostanoids are reserved for high-risk patients. Breakthroughs in PAH treatment in the recent years include the approval of the soluble guanylyl cyclase (sGC) stimulator riociguat and the oral prostacyclin receptor agonist selexipag. It is becoming increasingly apparent that by targeting multiple pathological pathways using combination therapy, we ensure the best outcome for our patients, as this strategy improves pulmonary hemodynamics, functional class, and cardiac functional parameters [87].

However, the direct effects of PAH-targeted therapy on the RV have not yet been sufficiently investigated. The Euro MR Study, based on MRI—which looked at patients from Glasgow (UK), Rome (Italy), Graz (Austria), and Amsterdam (the Netherlands) before and after 4 and 12 months—showed that the RV systolic and diastolic volumes as well as the SV improved after the initiation of PAH-specific therapy [88]. Few studies are published with data on RV function estimated by echocardiography in relation to PAH-targeted therapy. Table 1 presents human studies with PAH-targeted therapy and their effect on RV function based on echocardiographic parameters [78,89,90,91,92,93,94,95,96,97,98,99,100,101,102,103,104,105,106,107,108,109,110,111]. Both monotherapy and especially combination therapy significantly improve RA size, RV size, diastolic eccentricity index, MPI and TAPSE, RV strain, and RV strain rate, and cause greater alterations of the RV end-diastolic area and systolic and diastolic eccentricity index [37,112,113,114,115,116]. Up-front triple combination therapy in severe non-reversible PAH was associated with right heart remodeling and considerable improvement in RHC parameters [89].

## 5. Follow-Up in PAH: Repeated Assessment with RHC or Echocardiography?

There is a debate in the literature, and between experts, whether it is truly essential to perform serial RHCs for PAH patients’ follow-up, or whether non-invasive methods could provide accurate prognostic evaluation. RHC is an invasive procedure, not always available in all hospitals, and may infrequently lead to several complications—especially when performed by non-experts. On the other hand, echocardiography is a non-invasive procedure, with no potential harm, feasible in everyday clinical practice, and with less cost. Routine invasive follow-up may not be necessary in patients with low-risk non-invasive criteria [76,117]. Moreover, progressive RV dysfunction may not be accompanied by hemodynamic changes, such as in PVR. In a cohort of 110 patients, Van der Veerdonk et al. found that after the initiation of PAH-targeted therapy, RV function may deteriorate despite a reduction in PVR [20]. In this cohort, changes in PVR did not differ between survivors and non-survivors; however, during follow-up, survivors showed increased RVEF, whereas non-survivors showed decreased RVEF, suggesting that loss of RV function is associated with a poor outcome, irrespective of any changes in PVR. Another important finding is that signs of RV deterioration can be seen in PAH patients with no evidence of clinical deterioration. Van der Veerdonk et al. included 22 stable idiopathic PAH patients (as reflected by stable or improving New York Heart Association functional class II–III and exercise capacity) and performed a 5-year follow-up. RV volumes and RVEF seemed to deteriorate in some stable patients, and changes in these parameters could precede disease progression and mortality [118].

The additive benefit of RV function—as assessed by echocardiography, on top of well-established risk prediction models—is not thoroughly studied. Haddad et al. constructed a right heart score—which incorporated RV systolic function grade, severe RA enlargement, and systemic blood pressure < 110 mmHg—and this compared favorably with the NIH survival equation, and did not differ from the REVEAL score, while it was also the only predictor of outcome in the validation cohort [119]. In addition, the RV end-systolic remodeling index (defined by the ratio of the lateral RV wall length to the septal height) was incremental to predictive risk models, including the REVEAL score [120]. The RV free wall LSS and the right atrial peak longitudinal strain have also been shown to have an additive prognostic value and improve risk stratification in incident, naïve to PAH-targeted therapies [121]. Recently, Ghio et al. have evaluated an echocardiographic approach based on multiple parameters (TAPSE, tricuspid regurgitation, and inferior vena cava diameter) to separate PAH patients into three groups that represent progressive degrees of RV impairment [122]. They showed that this approach is effective in stratifying the probability of survival in the PAH population, while the inclusion of RA area and pericardial effusion did not add prognostic value to this approach. Lastly, Zhao et al. demonstrated that the echocardiographically measured attenuated right heart remodeling—as defined by the presence of decreased RA area, RV mid-diameter, and LV end-diastolic eccentricity index—was independently associated with mortality, and also increased the diagnostic ability of the French non-invasive risk assessment criteria [123].

## 6. Conclusions

**Figure 1:**
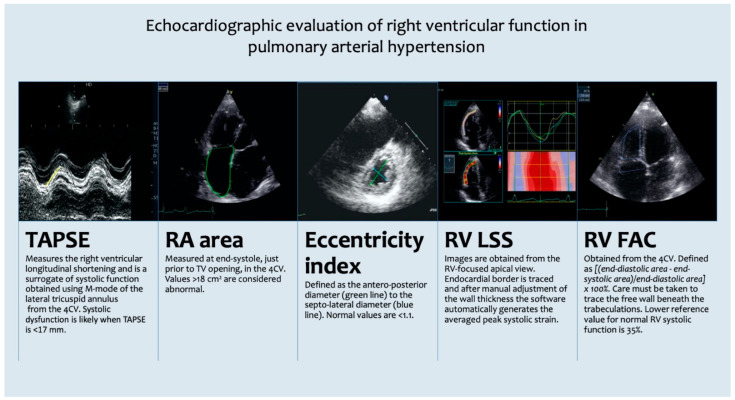
Echocardiographic evaluation of right ventricular function in pulmonary arterial hypertension. 4CV: four-chamber view; FAC: fractional area change; LSS: longitudinal free-wall strain; RA: right atrium; RV: right ventricular; TAPSE: tricuspid annular plane systolic excursion.

The awareness of the importance of the RV in PAH has increased considerably. Hypothetically, if normal RV function were the only goal during the follow-up of PAH patients, their survival would be better. Today, non-invasive imaging is increasingly being used for the study of RV and pulmonary circulation, with increased focus on functional relevance. Echocardiography is a widely accessible, non-invasive method, and provides a global assessment of RV function. An important question is how best to incorporate the accumulating data on echocardiographic parameters for the prognostic significance of PAH into investigation and clinical practice. According to the published data, it seems that TAPSE, RA area, RVFAC, eccentricity index, and RVLSS could be the echocardiographic parameters that we can use in our daily practice, to evaluate the RV function in PAH patients at baseline, and during their follow-up after specific drug therapy (Figure 1).

Further echocardiographic studies are needed to support these recommendations on the prognostic role of specific echocardiographic indices both at baseline, and especially during follow-up assessments, to evaluate the effect of PAH-targeted treatments on RV performance as measured by echocardiography. However, since echocardiography is not as reproducible or accurate as CMR, which seems to be the gold standard in the assessment of RV function, larger validation studies are needed in order to see which of those indices are most applicable to follow-up risk assessments. A large cohort outcome study comparing the afore-mentioned echocardiographic indices would also provide more answers as to whether the normalization of RV size and function as assessed by echocardiography predicts outcomes, either alone or in addition to multimodal parameters. Future trials of therapeutic interventions should incorporate echocardiographic indices as endpoints, and their association with clinical and hemodynamic parameters. Therefore, much work remains to take place identifying the most relevant indices for RV function and ventriculoarterial coupling estimation and their sensitivity to treatment strategies.

## Figures and Tables

**Table 1:** Human studies with PAH-targeted therapy and their effect on RV function based on echocardiographic parameters.

Authors	N of Patients	Treatment (Duration)	Effect on ECHO Parameters	Effect on Other Parameters
D’Alto et al. [89] [2020)	21	Ambrisentan + Tadalafil + Treprostinil (2 years)	↓RAA ↓RVA ↓LV EI ↑RVFAC	↑6MWD ↓RAP ↓mPAP ↓PVR ↓NT-proBNP ↑CI
Saggar et al. (2013) [90]	15	Treprostinil (12 weeks)	↓RVEDA ↓LV EI ↑TAPSE	↑6MWD ↓RAP ↓mPAP ↑CI ↓PVR ↓TPG ↓SVi ↓BNP
Mercurio et al. (2017) [91]	23 (Scleroderma patients)	Tadalafil + Ambrisentan (36 weeks)	↓RAA ↓RVA ↓RV FWT ↑RVFAC ↑TAPSE ↓RVSP ↓RVLSS	
Taran et al. (2018) [92]	20	Riociguat (12 weeks)	↓RAA ↓RV basal diameter ↑TAPSE ↓Diastolic EI ↑RV FAC ↓PASP ↓RVEDV ↓RVESV ↑RVEF ↓RV-PA coupling ↓PA Ea	↑6MWD ↑Peak VO2
Tonelli et al. (2014) [93]	48	Parenteral prostacyclin analogues (12 months)	↓RAA ↓RVA ↓TVR Vmax ↑TAPSE ↑RVOT VTI	
Nath et al. (2005) [94]	20	Epoprostenol (22.7 ± 9.3 months)	(−) RVMPI ↓TVR Vmax ↓PASP/PV VTI	
Hinderliter et al. (1997) [78]	81	Epoprostenol (12 weeks)	↓RVEDA ↓RV EI ↓TVR jet (−) PE (−) RVFAC	
Kaya et al. (2012) [95]	23 (Eisenmenger patients)	Bosentan (24 ± 9 months)	↓PASP ↓MPI ↑Sa ↑Ea	↑6MWD ↑Sat02
Kim et al. (2016) [96]	19	Bosentan (6 months)	↓RVSP ↑TAPSE ↑MPI (LV-RV) ↑RVFAC ↑RVEF	↑6MWD
Borges et al. (2006) [97]	37	Vasodilators (8 ± 3 months)	↓RV 2d-strain	
Galie et al. (2003) [98]	56	Bosentan (16 weeks)	↓RV ESA ↓RV EDA ↓LV EDA ↓LV EI ↑CI ↑LV early Diastolic filling pressures Improves PE score	↑6MWD
Ruiz et al. (2006) [99]	20	Prostanoids + Sildenafil * (2 years)	↓RVEDD ↓LVEI ↓RAA	↑6MWD
Jimenez Loper-Guarch et al. (2004) [100]	11	Prostacyclin + Sildenafil * (12 months)	↓RVEDD ↓LV EI	↑6MWD
Shat et al. (2015) [101]	202	Imatinib + 2 PAH specific Drugs (24 months)	↑TA S’ RV Tei ↑Index (−) TAPSE	
Gusca et al. (2012) [102]	32 (Eisenmenger patients)	Bosentan Sildenafil Bosentan + Sildenafil (14 months)	(−) RVFAC (−) RV global strain	
Hsu et al. (2007) [103]	15	Bosentan (12 months)	↑RVEF	↑6MWD ↓CTR
Hassoun et al. (2015) [104]	24 (Scleroderma patients)	Ambrisentan + Tadalafil (36 weeks)	(−) RVEDV ↓RVESV ↑RVEF ↑LVEDV ↑LVESV ↑TAPSE	↓RV mass ↓PVR ↓mPAP ↓RAP ↑CI ↑Sat O2 ↑NTproBNP ↑6MWD ↑SV/PP
Agapito et al. (2008) [105]	5 (Eisenmenger patients)	Iloprost Sildenafil Bosentan	↓RV-RA gr RV Tei ↓index	↑6MWD
Tacoy et al. (2014) [106]	12 (Eisenmenger patients)	Iloprost Sildenafil Bosentan (5 years)	↓PASP ↓RVFWT ↓PV ac t ↑RAA/LAA ↑RVFAC	↓RAP
Hansmann et al. (2020) [107]	15 (children)	Selexipag (8 months)	↑TAPSE	↓RAP ↓TPG ↓mPAP
Badagliacca et al. (2018) [108]	69	ERA PDE5i Prostanoids (155 ± 65 days)	↓RVEDA ↓RVESA ↑RVFAC ↑TAPSE ↑RAA ↓LVEDA ↓LVESA ↓LVEI	↑6MWD ↓RAP ↓mPAP ↑CI ↓PVR
Gabrielli et al. (2016) [109]	20	Iloprost	↑RVFAC ↓RV Dys in ↑RA res f	
Cha et al. (2013) [110]	18 (Eisenmenger patients)	Iloprost (24 weeks)	↓RVMPI (−) PASP	↑MWD (−) PVR
Marra et al. (2018) [111]	71	Riociguat (12 months)	↓RAA ↓RVA ↓RVFWT ↓TVR ↑TAPSE ↑RVFAC	

RAA: right atrium area; RVA: right ventricle area; LV EI: left ventricle eccentricity index; RVFAC: right ventricle fractional area; RVEDA: right ventricle end-diastolic area; TAPSE: tricuspid annular plane systolic excursion; RVFWT: right ventricle free-wall thickness; RVSP: right ventricle systolic pressure; RVLSS: right ventricle longitudinal systolic strain; EI: eccentricity index; PASP: pulmonary artery systolic pressure; RVEDV: right ventricle end-diastolic volume; RVESV: right ventricle end-systolic volume; RVEF right ventricle ejection fraction; RV: right ventricle; PA: pulmonary artery; TVR: tricuspid valve regurgitation; Vmax: maximal velocity; RVOT VTI: right ventricle outflow tract velocity time integral; RVMPI: right ventricle myocardial performance index; PVVTI: pulmonary valve velocity time integral; PE: pericardial effusion; Sa: systolic myocardial velocity; Ea: early diastolic myocardial relaxation velocity; RVESA: right ventricle end-systolic area; LVEDA: left ventricle end-diastolic area; LVESA: left ventricle end-systolic area; CI: cardiac index; RVEDD: right ventricle end-diastolic diameter; TA S’: tricuspid annular peak systolic velocity; LVEDV: left ventricle end-diastolic volume; LVESV: left ventricle end-systolic volume; RV Dys in: right ventricle dyssynchrony index; RA res f: right atrium reservoir function (speckle tracking); 6MWD: 6-min walking distance; RAP: right atrial pressure; mPAP: mean pulmonary artery pressure; PVR: pulmonary vasculature resistance; TPG: transpulmonary gradient; SVi: stroke volume index; Sat: saturation; SV: stroke volume; PP: pulmonary artery pulse pressure; (−): no effect. * Effect of added sildenafil.
